# Antioxidant and Pancreatic Lipase Inhibitory Activities of *Panax japonicus* (T. Nees) C.A. Meyer

**DOI:** 10.3390/plants14132003

**Published:** 2025-06-30

**Authors:** Jinfeng Yang, Wenxuan Jiang, Ju Hee Park, Eun Soo Seong, Yong Soo Kwon, Myong Jo Kim

**Affiliations:** 1 Research Institute of Food Science & Engineering Technology, Hezhou University, Hezhou 542899, China; 2 Guangxi Key Laboratory of Health Care Food Science and Technology, Hezhou University, Hezhou 542899, China; 3 College of Biological & Chemical Engineering, Guangxi University of Science and Technology, Liuzhou 545006, China; 4 Department of Applied Plant Sciences, Kangwon National University, Chuncheon 24341, Republic of Korea; 5 Department of Pharmacy, Kangwon National University, Chuncheon 24341, Republic of Korea; 6 Bioherb Research Institute, Kangwon National University, Chuncheon 24341, Republic of Korea

**Keywords:** obesity, antioxidant, *Panax japonicus* (T. Nees) C.A. Meyer, pancreatic lipase

## Abstract

Obesity and its associated complications, including oxidative stress, pose significant global health challenges. Natural products offer a promising avenue for developing novel therapeutic strategies. In this study, we investigated the potential of *Panax japonicus* (T. Nees) C.A. Meyer, a traditional medicinal plant known for its antioxidant and anti-obesity properties. A methanol extract of *Panax japonicus* and its fractions were evaluated for their in vitro antioxidant activities (tested using DPPH and reducing power assays), pancreatic lipase (PL) inhibitory capacities, and underlying mechanisms of action. The results indicated that the ethyl acetate fraction of *P. japonicus* (PJEA) exhibited the greatest potency, demonstrating strong antioxidant activity and significantly inhibiting digestive enzyme activity (pancreatic lipase). Mechanistic studies revealed that the PL inhibition was of a mixed type, combining both competitive and non-competitive mechanisms. Furthermore, PJEA demonstrated the ability to inhibit the differentiation of preadipocytes, primarily exerting its anti-adipogenic effects by downregulating the mRNA expression of PPARγ and the gene expression of C/EBPα. In addition, the extract suppressed the gene expression of FAS and ACC in adipose tissue. Isolation of the bioactive compounds from PJEA identified kaempferol 3-O-α-L-rhamnoside and catechin, which potentially contribute to the observed anti-obesity effects. Overall, this study highlights *P. japonicus* as a promising natural ingredient for scavenging free radicals and managing obesity, suggesting its potential for development into functional foods or therapeutic agents.

## 1. Introduction

Obesity and its associated complications, including metabolic disorders and oxidative stress, pose significant global health challenges [1,2]. The rapid increase in obesity prevalence is a major concern, with projections indicating a continuous rise in affected individuals worldwide. This metabolic disorder is characterized by dyslipidemia, which is marked by elevated levels of circulating lipids and cholesterol [3,4]. Pancreatic lipase (PL), a key enzyme in dietary lipid digestion, hydrolyzes triglycerides into absorbable fatty acids and monoglycerides [4]. Inhibiting PL activity is a recognized pharmacological strategy for preventing and managing obesity by impeding triglyceride absorption [5].

Adipocytes, the primary cellular components of adipose tissue, are crucial for lipid storage and systemic energy homeostasis. Abnormal adipocyte proliferation and differentiation contribute significantly to obesity pathogenesis and related metabolic disorders, such as type 2 diabetes and hyperlipidemia [6]. The 3T3-L1 cell line serves as a widely used in vitro model to study adipocyte differentiation, a process regulated by key transcription factors like the peroxisome proliferator-activated receptor gamma (PPARγ) and CCAAT/enhancer-binding protein α (C/EBPα) [7]. While pharmaceutical interventions like Orlistat exist to achieve PL inhibition, their widespread use is limited by adverse gastrointestinal effects. This necessitates the identification of safe, efficacious, and naturally derived bioactive compounds for obesity management [8].

Natural compounds, particularly plant polyphenol extracts, have gained considerable attention for their potential in treating obesity and lipid metabolism disorders through interactions with lipid-digesting enzymes. For example, citrus peel extracts, which are rich in phenolic constituents, have shown effectiveness in inhibiting pancreatic lipase, suggesting their possible importance for obesity management [9].

*Panax japonicus* (T. Nees) C.A. Meyer, a perennial herbaceous plant of the Araliaceae family, is traditionally valued in East Asian medicine [10]. Beyond its uses in folk medicine, contemporary pharmacological investigations have revealed its diverse bioactivities, including anti-inflammatory, immunomodulatory, and protective effects against various diseases. These effects are attributed to its rich composition of bioactive constituents, such as saponins, polysaccharides, amino acids, volatile oils, and trace elements [11,12].

Despite the plant’s traditional use and documented pharmacological activities, current research on extracts of *P. japonicus* with varying polarities remains limited, particularly concerning a comprehensive evaluation of their antioxidant potential and detailed effects on weight modulation. Specifically, there is a scarcity of studies investigating the specific mechanisms by which different fractions of *P. japonicus* influence pancreatic lipase activity and adipocyte differentiation at a molecular level, or the identification of the specific bioactive compounds responsible for these effects.

Therefore, this study aims to comprehensively evaluate the antioxidant activities and pancreatic lipase inhibitory capacities of a methanol extract of *P. japonicus* and its various polarity fractions. Building on these findings, we will conduct in vitro enzyme kinetics experiments to elucidate the mode and mechanism of pancreatic lipase inhibition. Furthermore, by utilizing the 3T3-L1 preadipocyte model, coupled with Oil Red O staining and the assessment of key differentiation markers, we will detail the molecular mechanisms by which the extracts modulate adipocyte differentiation. Finally, an activity-guided fractionation strategy will be employed to facilitate the tracking, isolation, and structural characterization of the bioactive constituents responsible for the observed anti-obesity effects. This study seeks to provide a novel theoretical foundation and empirical support for the valorization of *P. japonicus* resources and the development of functional food ingredients with hypolipidemic and antioxidant properties.

## 2. Results and Discussion

### 2.1. Comparison of Polyphenol Extraction Methods from P. japonicus

The yield of the extracted product is a critical parameter that significantly influences the industrial scalability and economic viability of the process. As shown in Table 1, the extraction yields obtained from the four different solvents applied to *P. japonicus* ranged from a minimum of 29.31% to a maximum of 50.12%. The order of extraction yields, from highest to lowest, was as follows: methanol extract > water extract > ethanol extract > acetone extract. Additionally, the data in Table 1 reveal that all four extracts of *P. japonicus* contained substantial amounts of total phenolic compounds. The extraction efficiency of total phenolic content (TPC), ranked from highest to lowest, aligned with the overall extraction yield pattern: methanol extract > water extract > ethanol extract > acetone extract. This trend may be due to a combination of factors, including solvent polarity and the complex nature of phenolic compounds. While ethanol may not effectively disrupt the interactions between phenolic compounds and other cellular constituents, methanol and water, having higher polarity indices, show a greater ability to dissolve a wider range of phenolic compounds. Variabilities in the chemical properties of phenolic compounds such as dipole moments, their hydrogen bonding capabilities, and their polarizability influence their solubility in solvents with different polarities [13]. Thus, the selective affinity of solvents for specific types of compounds based on their polarity results in variations in the overall extraction yield and the extracted phenolic content.

In addition to the total phenolic content, the total flavonoid content (TFC) of each extract was also evaluated using the aluminum chloride colorimetric method. As shown in Table 1, the methanol extract exhibited the highest TFC (48.02 mg QE/g), followed by the ethanol and water extracts. The acetone extract yielded the lowest TFC (16.67 mg QE/g). This distribution trend was generally consistent with that of TPC and antioxidant activity, indicating that both phenolic and flavonoid compounds are key contributors to the antioxidant potential of *P. japonicus*.

The 2,2-diphenyl-1-picrylhydrazyl (DPPH·) radical scavenging activity varied among the *P. japonicus* extracts obtained with different solvents. Notably, the methanol extract demonstrated the most potent scavenging activity, with an IC_50_ value of 36.33 μg/mL, followed by the water extract (IC_50_ = 40.47 μg/mL). The ranking of DPPH· radical scavenging activity, from highest to lowest, was as follows: methanol extract > water extract > ethanol extract > acetone extract. These findings indicate that the *P. japonicus* methanol extract exhibits significant radical scavenging potential, which correlates well with its high total phenolic content. Given its superior extraction yield, the highest measured concentration of total phenolic compounds, and the most robust antioxidant activity, the methanol extract was chosen for further detailed experimental analysis to elucidate its bioactivity properties. However, it is important to acknowledge that while methanol is highly effective for laboratory-scale extraction, its inherent toxicity limits its direct application in the food or pharmaceutical industries. Therefore, future research will explore alternative, food-grade solvents or advanced extraction techniques to achieve comparable efficacy for industrial and practical applications.

### 2.2. Antioxidant Activity of P. japonicus

To assess the antioxidant potential of *P. japonicus* further, the crude methanol extract was sequentially partitioned using solvents of increasing polarity: hexane, ethyl acetate, n-butanol, and distilled water. As indicated in Table 2, the total phenolic content (TPC) and total flavonoid content (TFC) displayed significant variations across these different polarity fractions. Notably, the ethyl acetate (EtOAc) fraction exhibited the highest levels of both TPC and TFC, reaching 198.51 mg GAE/g and 68.63 mg QE/g, respectively. The n-butanol fraction was the second-richest in both phenolic and flavonoid compounds. Conversely, the hexane and water fractions showed the lowest TPC and TFC, which were even below those observed in the crude methanol extract. This distribution suggests that phenolic and flavonoid compounds, known for their antioxidant contribution, are predominantly concentrated in the moderately polar ethyl acetate fraction of the *P. japonicus* methanol extract. As also shown in Table 2, the capacity of the different polarity fractions of *P. japonicus* to scavenge DPPH radicals varied considerably. The *n*-butanol fraction demonstrated potent DPPH radical scavenging activity at higher concentrations, yielding an IC_50_ value of 65.31 μg/mL. The ethyl acetate fraction, however, exhibited the most potent scavenging ability at lower concentrations, with a notably lower IC_50_ value of 5.39 μg/mL. In contrast, the water fraction exhibited the weakest DPPH radical scavenging activity, with an IC_50_ value of 123.87 μg/mL. These disparities highlight the variability in antioxidant compound distribution among the different polarity fractions of the *P. japonicus* methanol extract.

**Figure 1:**
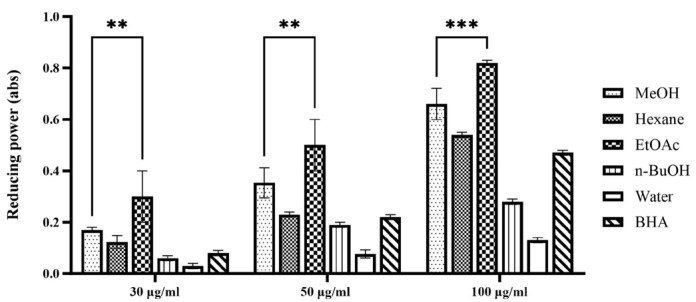
Reducing power assay of extract and solvent fractions from *P. japonicus*. The figure illustrates the dose-dependent reducing power of various extracts (methanol (MeOH), hexane, ethyl acetate (EtOAc), n-butanol (n-BuOH), water) and a positive control (BHA) from *P. japonicus* at concentrations of 30 µg/mL, 50 µg/mL, and 100 µg/mL. Higher absorbance values at 734 nm indicate greater reducing power. Statistical significance is denoted as ** *p* < 0.01 and *** *p* < 0.001, comparing the ethyl acetate fraction to the water fraction at each concentration, demonstrating its significantly higher reducing capacity.

The ferric reducing antioxidant power (FRAP) assay quantifies the ability of a sample to reduce ferric ions (Fe^3+^) to ferrous ions (Fe^2+^). This assay primarily reflects electron transfer capability, a key mechanism through which antioxidants neutralize free radicals. Consequently, the FRAP assay offers complementary information alongside the DPPH radical scavenging assay, enabling a comprehensive evaluation of antioxidant capacity and elucidating the underlying mechanisms of action of the samples [14]. The results of the FRAP assay are expressed as absorbance values at 734 nm, with higher absorbance indicating a greater production of Fe^2+^ and, thus, enhanced reducing power and antioxidant capacity. As depicted in Figure 1, all tested extracts and fractions of *P. japonicus* exhibited a concentration-dependent reducing power within the examined range, demonstrating a progressive increase in reducing capacity with ascending concentrations. This trend is revealed by the order of ferric reducing antioxidant power (FRAP), which is as follows: ethyl acetate fraction > methanol extract > n-butanol fraction > water fraction. This pattern aligns with the outcomes from the DPPH radical scavenging activity assays, further asserting that the ethyl acetate fraction possesses significant antioxidant potential. Extensive research has shown that extracts derived using moderately polar solvents often display marked benefits in systems used to evaluate antioxidant activity. In the DPPH radical scavenging assay, the ethyl acetate fraction of Calendula officinalis exhibited a half-maximal inhibitory concentration (IC_50_) of 18.7 ± 0.5 μg/mL, significantly lower by over 50% compared to the n-butanol fraction (IC_50_ = 32.4 ± 0.8 μg/mL) and the water fraction (IC_50_ > 100 μg/mL), indicating a robust dose-dependent response [15]. Furthermore, the ethyl acetate extract of Areca catechu showed impressive scavenging rates of (92.3 ± 1.2)% for DPPH radicals and (89.7 ± 1.5)% for ABTS radicals, significantly exceeding those of the synthetic antioxidant butylated hydroxytoluene (BHA) (*p* < 0.01) [16]. The results from the ferric reducing antioxidant power (FRAP) assay further confirm this observation. The ethyl acetate fraction of Mentha haplocalyx displayed a ferrous ion reduction capacity of (4.73 ± 0.15) mmol FeSO_4_/g, which was 3.2 times higher than that of the petroleum ether fraction [17]. Additionally, the half-maximal inhibitory concentration for the iron ion reduction capacity of the ethyl acetate fraction of Tetrastigma hemsleyanum was only 0.352 mg/mL, emphasizing its effective electron transfer capability [18]. These literature precedents corroborate the findings of the current study, reinforcing the significant antioxidant potential associated with the moderately polar ethyl acetate fraction of *P. japonicus*.

### 2.3. Pancreatic Lipase Inhibitory Activity of P. japonicus Ethyl Acetate Fraction

**Figure 2:**
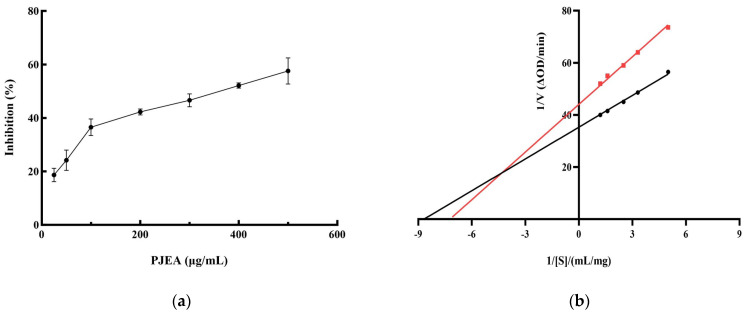
Inhibition rate and Lineweaver–Burk analysis of PJEA against pancreatic lipase. (**a**) Inhibitory effect of PJEA on pancreatic lipase, (**b**) The double-reciprocal (Lineweaver–Burk) plots illustrate the effect of PJEA on the kinetics of pancreatic lipase activity. The red squares represent data obtained in the presence of PJEA, while the black circles represent data obtained in the absence of PJEA.

Given the crucial role of pancreatic lipase (PL) in the digestion and absorption of dietary lipids, inhibiting its activity represents a functional strategy for the management of obesity. Therefore, we conducted further research into the ability of the ethyl acetate fraction (PJEA) to inhibit PL activity. As illustrated in Figure 2a, within the tested concentration range, PJEA demonstrated a robust and dose-dependent inhibitory effect on pancreatic lipase activity. The half-maximal inhibitory concentration (IC_50_) of PJEA for pancreatic lipase was determined to be 317 μg/mL. To elucidate the mechanism by which PJEA inhibits pancreatic lipase, enzyme kinetic analysis was performed. A Lineweaver–Burk plot was constructed by plotting the reciprocal of the substrate concentration (1/[S]) against the reciprocal of the initial reaction velocity (1/[V]), as depicted in Figure 2b. The resulting plot revealed that the lines obtained in the presence and absence of the inhibitor intersected in the second quadrant, indicating mixed inhibition. This type of inhibition, which involves both competitive and non-competitive mechanisms, illustrates that PJEA may compete with substrate molecules for binding to the enzyme’s active site, thereby reducing enzymatic catalytic activity, and may bind to a distinct site, causing a conformational change that further diminishes the enzyme’s function [9].

### 2.4. Effect of PJEA on the Proliferation of 3T3-L1 Preadipocytes

**Figure 3:**
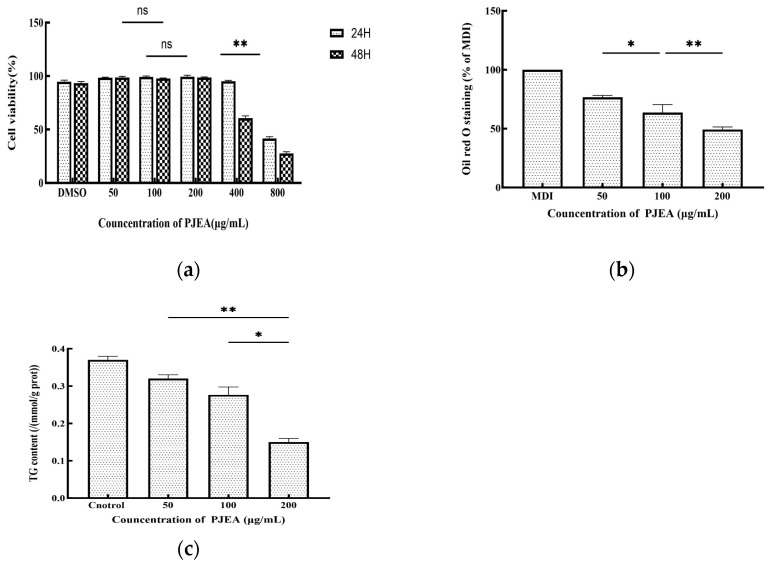
(**a**) Effect of PJEA on cell viability in 3T3-L1 cells following treatment with various concentrations (50, 100, and 200 μg/mL) of PJEA; (**b**) the effect of PJEA on the lipid accumulation seen in 3T3-L1 cells, with the inhibitory effect of PJEA on lipid accumulation in 3T3-L1 adipocyte differentiation tested using Oil Red O staining; (**c**) the effect of PJEA on TG accumulation in 3T3-L1 adipocyte differentiation. Each value denotes the mean ± standard deviation (SD) of three independent experiments (*n* = 3) (* *p* < 0.05, ** *p* < 0.01), ns: not significant.

The impact of PJEA on the proliferation of 3T3-L1 preadipocytes was evaluated by incubating the cells with varying concentrations of PJEA for 24 and 48 h. Cell viability was determined by comparing the optical density (OD) values of the treated groups with those of the control group. The results are presented in Figure 3a. Dimethyl sulfoxide (DMSO), which was used as a cosolvent to dissolve the extract, exhibited minimal cytotoxicity at the concentrations used, after both 24 and 48 h of incubation. The survival rate of 3T3-L1 preadipocytes demonstrated a dose-dependent response to PJEA across the range, tested from 50 μg/mL to 800 μg/mL. Particularly when the concentration of PJEA was increased from 50 μg/mL to 200 μg/mL, the survival rate remained above 90% after both 24 and 48 h, indicating minimal cytotoxicity at these lower concentrations. After 24 h of exposure, a statistically significant decline in cell survival at a concentration of 800 μg/mL was observed. Similarly, after 48 h of treatment, cell survival significantly decreased at 400 μg/mL. Based on these cytotoxicity findings, the final concentrations of PJEA selected for subsequent experiments to investigate its effects on adipocyte differentiation were 50 μg/mL, 100 μg/mL, and 200 μg/mL, as these concentrations exhibited minimal cytotoxicity while permitting the evaluation of potential biological effects.

### 2.5. Effect of PJEA on 3T3-L1 Cell Differentiation

To induce differentiation in the 3T3-L1 cells and generate lipid droplets, a differentiation medium consisting of DMEM supplemented with IBMX, DEX, and INS was utilized. Following a 10-day incubation period, the differentiated cells were subjected to Oil Red O staining. The intensity of the red color in the extraction solution correlated directly with the quantity of accumulated lipid droplets. As anticipated, the MDI differentiation medium (containing 0.5 mM 3-isobutyl-1-methylxanthine, 10 μg/mL insulin, and 1 μM dexamethasone) resulted in a red extraction solution, which was indicative of lipid droplet formation. To investigate the anti-adipogenic effects of PJEA on adipocyte differentiation, the 3T3-L1 cells were treated with PJEA at concentrations of 50, 100, and 200 μg/mL. After 10 days, PJEA treatment markedly and dose-dependently suppressed total lipid accumulation during the differentiation process. Specifically, treatment with 200 μg/mL of PJEA resulted in a reduction of over 51% in total lipid accumulation when compared to the control group (Figure 3b).

### 2.6. Effect of PJEA on Intracellular Triglyceride Levels in 3T3-L1 Adipocytes

To further elucidate the impact of PJEA on lipid metabolism in 3T3-L1 adipocytes, we quantified the intracellular triglyceride (TG) content. On the 10th day of differentiation, after treating the cells with varying concentrations of PJEA, they were harvested and analyzed. The results of this analysis are presented in Figure 3c; they demonstrated a dose-dependent decrease in intracellular triglyceride (TG) levels in 3T3-L1 cells with increasing concentrations of PJEA. Statistically significant reductions in TG levels were observed across all PJEA-treated groups in comparison to the control group, indicating an inhibitory effect of PJEA on lipid accumulation. Obesity, a state of energy imbalance wherein energy intake exceeds energy expenditure, culminates in the excessive accumulation of adipose tissue throughout the body. The expansion of adipose tissue mass can arise from both an increase in the number of differentiated adipocytes (hyperplasia) and an enlargement of the individual adipocyte volume due to augmented lipid storage (hypertrophy). Consequently, the inhibition of adipocyte proliferation and differentiation has emerged as a potential therapeutic strategy for the prevention and treatment of obesity and its associated metabolic disorders [19]. In recent years, a growing body of research has underscored the significant role of plant-derived polyphenols in ameliorating lipid metabolism disorders by modulating the intricate processes of adipocyte differentiation [20]. The 3T3-L1 preadipocyte cell line, which exhibits a robust capacity to recapitulate the stages of adipocyte differentiation and lipid accumulation in vitro, has become a widely utilized and representative cellular model for evaluating the anti-obesity effects of various functional components [21]. A series of studies employing this model have demonstrated the anti-adipogenic potential of various polyphenolic compounds. For instance, epigallocatechin gallate (EGCG), expressed in concentrations ranging from 50 μM to 200 μM, reduced intracellular triglyceride (TG) content in differentiated 3T3-L1 cells by 40–75%, with the 200 μM treatment group exhibiting lipid droplet volumes, as quantified by Oil Red O staining, at only 25% of those in the control group [22]. Similarly, curcumin, at a concentration of 20 μM, decreased the TG content in 3T3-L1 cells by 60% while concurrently inhibiting cell proliferation [23]. Furthermore, research by Uner et al. indicated that chlorogenic acid, used at concentrations of 5 μg/mL to 20 μg/mL, significantly reduced lipid droplet formation, with the 20 μg/mL treatment group showing a 48% reduction in TG content compared to the control group (*p* < 0.01) [24]. The preliminary findings of the present study are consistent with these aforementioned results, indicating a dose-dependent reduction in intracellular TG content in 3T3-L1 cells upon treatment with PJEA. Specifically, increasing concentrations of PJEA were associated with a gradual decrease in TG accumulation, suggesting that the *P. japonicus* extract may possess potential inhibitory effects on lipid accumulation in adipocytes. Building upon these initial findings, subsequent investigations within this study will delve deeper into the regulatory effects of the PJEA fraction on the molecular mechanisms governing lipid metabolism during adipocyte differentiation.

### 2.7. Effect of PJEA on the Expression of the PPARγ, C/EBPα, FAS, and ACC Genes in 3T3-L1 Preadipocytes

**Figure 4:**
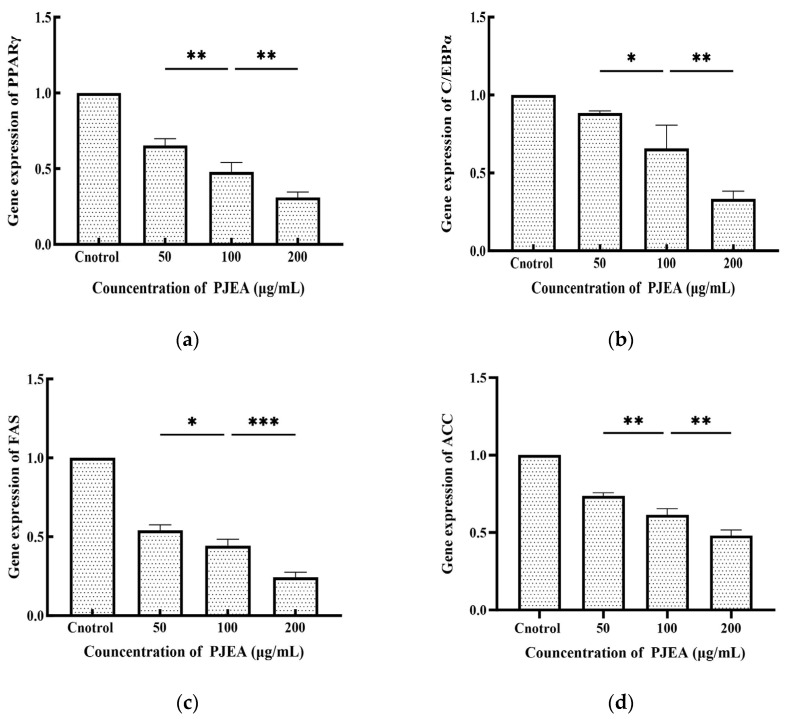
Effect of PJEA on genes in 3T3-L1 preadipocytes: (**a**) Effect of PJEA on the relative expression of PPARγ genes in 3T3-L1 preadipocytes; (**b**) Effect of PJEA on the relative expression of C/EBPα genes in 3T3-L1 preadipocytes; (**c**) Effect of PJEA on the relative expression of FAS genes in 3T3-L1 preadipocytes; (**d**) Effect of PJEA on the relative expression of ACC genes in 3T3-L1 preadipocytes (* *p* < 0.05, ** *p* < 0.01, and *** *p* < 0.001).

The CCAAT/enhancer-binding protein α (C/EBPα) and peroxisome proliferator-activated receptor γ (PPARγ) are crucial transcriptional regulators that oversee the differentiation of 3T3-L1 preadipocytes into mature adipocytes. As shown in Figure 4, PJEA treatment led to a substantial downregulation of C/EBPα gene expression at concentrations ranging from 50 μg/mL to 200 μg/mL compared to the untreated control group. A pronounced dose-dependent effect was evident, with increasing concentrations of PJEA resulting in progressively lower levels of C/EBPα expression. Figure 4 also demonstrates the effect of PJEA on PPARγ gene expression. Similarly, PJEA significantly reduced PPARγ gene expression relative to the control group, and this downregulation followed a dose-response pattern, with higher concentrations leading to decreased PPARγ expression. Fatty acid synthase (FAS) and acetyl-CoA carboxylase (ACC) play essential roles in the de novo lipogenesis pathway and significantly contribute to fat accumulation within adipocytes. As depicted in Figure 4, treatment with PJEA notably decreased FAS gene expression compared to the untreated control group, showing a clear dose-dependent effect, with increasing concentrations of PJEA resulting in lowered FAS mRNA levels. Adipogenesis, the transformation of preadipocytes into mature adipocytes, is strictly regulated by a sequence of gene expression modifications controlled by key transcription factors. Among these, the peroxisome proliferator-activated receptor γ (PPARγ) and CCAAT/enhancer-binding protein α (C/EBPα) serve as central regulators, which are essential for expressing multiple genes that are critical for adipocyte differentiation and lipid storage [25]. The necessity of PPARγ for adipogenesis is well-established; its absence prevents adipocyte differentiation [26]. Our results indicate that PJEA significantly suppresses the mRNA expression of PPARγ in a dose-dependent manner in 3T3-L1 preadipocytes. This finding implies a primary mechanism through which PJEA exerts its anti-adipogenic effects by inhibiting PPARγ expression and, thus, disrupting the complete adipogenic program, impairing the expression of the downstream target genes necessary for the full differentiation and functionality of adipocytes.

Furthermore, C/EBPα, another crucial transcription factor within the same pathway as PPARγ that is reliant on PPARγ signaling [27], was significantly downregulated by PJEA in a dose-dependent manner. This observation provides additional insight into the extract’s anti-adipogenic mechanism. The suppression of C/EBPα expression likely enhances the inhibitory effects recorded on adipocyte differentiation, which is vital for later stages of adipogenesis and the maintenance of the differentiated state. The concurrent downregulation of both pivotal transcription factors, PPARγ and C/EBPα, highlights PJEA’s potential to effectively target the core regulatory machinery of adipocyte differentiation. Beyond these master regulators, the expression of enzymes directly involved in lipid synthesis is critical for adipocyte maturation and lipid accumulation. Fatty acid synthase (FAS) and acetyl-CoA carboxylase (ACC), crucial enzymes in the de novo lipogenesis pathway, generally see increased expression during adipocyte differentiation, which significantly contributes to fat deposition [28]. Our analysis shows that PJEA significantly downregulates the mRNA expression of both FAS and ACC in a dose-dependent manner. It suggests that apart from inhibiting the differentiation process, the extract also suppresses the expression of key enzymes involved in synthesizing fatty acids, the building blocks of triglycerides, which are stored in lipid droplets. This dual mechanism of inhibiting differentiation and suppressing lipogenesislikely contributes to the observed reduction in intracellular triglyceride levels in 3T3-L1 adipocytes treated with PJEA.

### 2.8. Inhibition of Pancreatic Lipase Activity by Isolated Flavonoid Compounds

**Figure 5:**
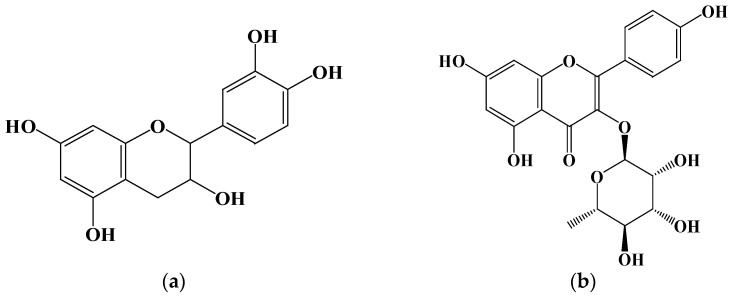
Structure of compounds from *P. japonicus*: (**a**) Compound 1: catechin; (**b**) Compound 2: kaempferol 3-O-α-L-rhamnoside.

**Figure 6:**
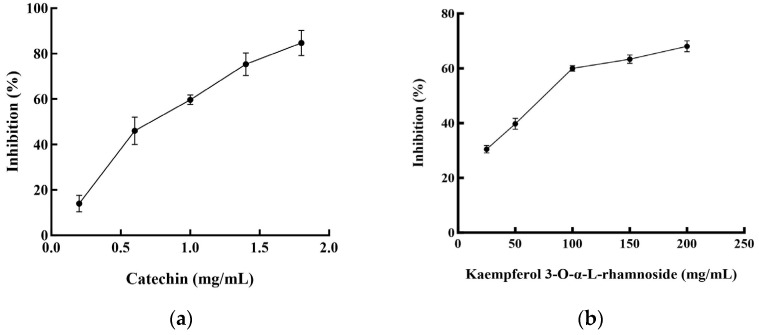
Pancreatic lipase inhibitory activities of the compounds from *P. japonicus*: (**a**) Pancreatic lipase inhibitory activities of catechin; (**b**) Pancreatic lipase inhibitory activities of kaempferol 3-O-α-L-rhamnoside.

Two flavonoid compounds, catechin and kaempferol 3-O-α-L-rhamnoside, were isolated from PJEA. Their chemical structures were confirmed using ESI-MS and NMR spectroscopic analyses (Figure 5), and the spectral data thus obtained were consistent with previously published findings [29]. To further explore the potential of the isolated flavonoids, catechin and kaempferol 3-O-α-L-rhamnoside, in modulating pancreatic lipase (PL) activity, a detailed investigation of their individual inhibitory effects on PL was conducted. The outcomes depicted in Figure 6a (for catechin) and Figure 6b (for kaempferol 3-O-α-L-rhamnoside) showed a clear concentration-dependent increase in PL inhibitory activity for both compounds. Notably, catechin demonstrated considerably more potent PL inhibition, with an IC_50_ value of 0.74 ± 0.001 mg/mL, whereas kaempferol 3-O-α-L-rhamnoside displayed inhibitory activity, albeit with a significantly higher IC_50_ value of 65.31 ± 0.021 mg/mL. This finding aligns with prior research that underscored the effectiveness of catechin as a potent inhibitor of pancreatic lipase [30].

Plant-derived foods are rich sources of secondary metabolites, including polyphenols, saponins, and terpenes, with flavonoids representing the most abundant polyphenolic compounds in human diets [31]. Characterized by a conserved three-ring (C6-C3-C6) structure, flavonoids exhibit diverse structural variations that classify them into subclasses such as flavones (including flavonols), flavanones, isoflavones, flavan-3-ols, and anthocyanins. Accumulating research data underscore the potential of specific flavonoids to inhibit lipase activity, a critical target in obesity management [32]. For instance, quercetin and galangin have shown both in vitro and in vivo inhibitory effects on pancreatic lipase (PL), while tea polyphenols, including EGCG, have also shown promise. Additionally, flavonoids such as vitexin have been implicated in anti-adipogenic mechanisms [33]. In this study, we isolated two flavonoid compounds, catechin (a flavan-3-ol) and kaempferol 3-O-α-L-rhamnoside (afzelin, a flavonol glycoside), from the *Panax japonicus* ethyl acetate (PJEA) fraction. Catechin, found in sources like Senegalia catechu and Camellia sinensis, is known for its wide range of biological activities, including potential anti-obesity effects [34]. Studies in obese mice have suggested the efficacy of catechin in reducing body fat and regulating lipid metabolism, and in vitro studies have confirmed its inhibitory effect on human pancreatic lipase, positioning it as a safer alternative to orlistat [35]. Kaempferol, a prevalent flavonoid in various foods, and its derivatives, including kaempferol 3-O-α-L-rhamnoside (afzelin), have also demonstrated potential benefits for metabolic disorders. Peng et al. identified the compound in plants like Smilax china and Bauhinia variegata and showed it to have significant in vitro PL inhibitory activity [36]. Importantly, Li et al. elucidated the molecular interaction between afzelin and PL, demonstrating its direct binding to the active site, leading to competitive inhibition [37]. Our findings reinforce these previous studies as both the isolated compounds, catechin and kaempferol 3-O-α-L-rhamnoside, exhibited significant pancreatic lipase inhibitory activity. Catechin displayed a markedly lower IC_50_ value (0.74 ± 0.001 mg/mL) compared to kaempferol 3-O-α-L-rhamnoside (65.31 ± 0.021 mg/mL), indicating that it is a more potent PL inhibitor. The presence of hydroxyl groups on the C-ring and the B-ring of both catechin and kaempferol likely facilitates their interaction with PL through hydrogen bonding and hydrophobic interactions, enhancing their inhibitory potential [38]. The higher solubility of catechin, attributed to its B-ring hydroxyl groups, may further enhance its interaction with PL, explaining its stronger inhibitory effect. Overall, the isolation of catechin and kaempferol 3-O-α-L-rhamnoside from the *P. japonicus* extract and their demonstrated PL inhibitory activity furnish a mechanistic basis for the anti-obesity potential of this plant. These findings further corroborate the increasing evidence that plant-derived flavonoids constitute a valuable source of natural compounds for managing lipid metabolism and preventing obesity.

## 3. Materials and Methods

### 3.1. Preparation of P. japonicus Extract

The dried *P. japonicus* powder was separately macerated in 100 mL of methanol, ethanol, acetone, and distilled water for a 24-h period. Subsequently, the resulting suspensions were filtered to obtain the respective filtrates. The remaining residue from each solvent extraction was subjected to three additional extraction cycles using the same solvent, and the resulting filtrates were pooled. Each combined filtrate was then concentrated under reduced pressure, using a rotary evaporator for subsequent utilization.

### 3.2. DPPH Radical Scavenging Activity Assay

The antioxidant capacity of the *P. japonicus* extracts was evaluated using the 2,2-diphenyl-1-picrylhydrazyl (DPPH·) radical scavenging assay, following a slightly modified version of a previously established method [39]. Briefly, a 0.3 mM solution of DPPH· in methanol was prepared. Different concentrations of each extract were prepared in the corresponding extraction solvent and then reacted with the DPPH· solution. For each assay, 100 μL of the extract solution was mixed with 100 μL of the DPPH· solution in a 96-well microplate. The mixture was incubated in the dark at room temperature for 30 min. The absorbance was then measured at 517 nm using a microplate reader (Thermo Scientific Multiskan GO, Waltham, MA, USA). Butylated hydroxyanisole (BHA) was used as a positive control for benchmarking antioxidant activity. Methanol was used as a blank, and a DPPH· solution without the extract served as the control. The DPPH· radical scavenging activity was calculated using the following equation: Scavenging activity (%) = [(Abs control − Abs sample)/Abs control] × 100 (Abs control = Absorbance of the DPPH control (without sample); Abs sample = Absorbance of the sample mixture). The concentration at which the scavenging activity is 50% is known as the IC_50_ value.

### 3.3. Preparation and Isolation of Biological Compounds from P. japonicus Methanol Extract

The dried and powdered *P. japonicus* samples (300 g) were subjected to maceration in 8 L of methanol at a solid-to-solvent ratio of 1:20 (*w*/*v*) for 72 h. The resulting suspension was then filtered under a vacuum to obtain the initial methanol extract. The marc (the remaining solid residue) was subsequently re-extracted three additional times with the same volume of methanol, and the resulting filtrates were combined with the initial extract. This combined methanol extract was concentrated under reduced pressure using a rotary evaporator until a viscous paste was obtained. A predetermined quantity of the methanol extract was then reconstituted in 500 mL of distilled water, and sequential liquid–liquid extractions were performed using solvents of increasing polarity: hexane, ethyl acetate, and *n*-butanol. Each solvent was used for four to five successive extractions to ensure maximal solute recovery. After sequential liquid–liquid extractions and concentrations, the yields for the fractions were obtained as follows: hexane fraction (12.31 g), ethyl acetate fraction (58.63 g), *n*-butanol fraction (16.29 g), and aqueous fraction (81.95 g). The ethyl acetate (EtOAc) extract (58.63 g) was subjected to primary fractionation using silica gel column chromatography (200–270 mesh). The column was eluted with a gradient solvent system of benzene:ethyl acetate:methanol, starting with a ratio of 80:10:10 (*v*/*v*/*v*) and gradually progressing to 0:0:100 (*v*/*v*/*v*) to yield four major fractions (Fr.1-Fr.4). Fraction 4 was further purified by silica gel column chromatography employing a gradient elution system of chloroform:methanol, ranging from 100:0 (*v*/*v*) to 0:100 (*v*/*v*), resulting in the collection of fourteen sub-fractions (Fr.4-1 to Fr.4-14). Sub-fraction Fr.4-12 was then subjected to reversed-phase octadecylsilyl (ODS) column chromatography for final purification. The column was eluted with a gradient of water:methanol, commencing at 90:10 (*v*/*v*) and progressing to 0:100 (*v*/*v*), which led to the isolation of two compounds: compound **1** (134.7 mg) and compound **2** (83.6 mg). The resulting hexane, ethyl acetate, *n*-butanol, and aqueous extracts, as well as isolated compounds (compound **1** and compound **2**), were stored at −20 °C in airtight, amber glass vials and protected from light for the duration of the study to maintain their stability for subsequent experimentation.

### 3.4. Analysis of Total Phenolic Content

The total phenolic content (TPC) was determined using the Folin–Ciocalteu reagent method, following a previously described protocol with slight modifications [40]. Briefly, 0.1 mL of the diluted sample was reacted with 0.05 mL of Folin–Ciocalteu reagent (Sigma-Aldrich, F9252, St. Louis, MO, USA) in a test tube. After a 4-minute incubation period at room temperature, 0.3 mL of a 20% (*w*/*v*) sodium carbonate (Na_2_CO_3_) solution was added to the reaction mixture, and the contents were immediately vortexed to ensure thorough mixing. Subsequently, 1.0 mL of distilled water was added, and the final mixture was incubated at room temperature in the dark for 15 min. The absorbance of the resulting blue-colored solution was measured at 725 nm using a UV-Vis spectrophotometer (Shimadzu UV-1800, Kyoto, Japan). All measurements were performed in triplicate. The total phenolic content was expressed as milligrams of gallic acid equivalents per gram of extract (mg GAE/g) and calculated by interpolating the absorbance values against a previously established calibration curve, prepared using standard gallic acid solutions.

### 3.5. Determination of Total Flavonoid Content

The total flavonoid content (TFC) was determined using the method described by Ardestani et al. [41], with slight modifications. For the assay, the 0.5 mL sample was reacted with 0.15 mL of a 15% (*w*/*v*) sodium nitrite (NaNO_2_) solution. After a 6-minute incubation period at room temperature, 0.15 mL of a 10% (*w*/*v*) aluminum chloride (AlCl_3_) solution was added, and the mixture was incubated for another 6 min at room temperature. Subsequently, 2.0 mL of a 4% (*w*/*v*) sodium hydroxide (NaOH) solution was added, and the total volume of the reaction mixture was adjusted to 5.0 mL by the addition of distilled water. After thorough mixing, the solution was allowed to react at room temperature for 15 min, and the absorbance was measured at 510 nm using a UV-Vis spectrophotometer (Shimadzu UV-1800).

### 3.6. Reducing Power Assay

The reducing power of the *P. japonicus* extracts was determined according to a previously described method [42], with minor modifications. Briefly, different concentrations of each extract were prepared in distilled water. To each concentration, 2.5 mL of a 0.2 M phosphate buffer (pH 6.6) and 2.5 mL of a 1% potassium ferricyanide [K_3_Fe(CN)_6_] solution were added. The mixture was then incubated at 50 °C for 20 min in a water bath. Following incubation, 2.5 mL of a 10% trichloroacetic acid (TCA) solution was added to the mixture, and the resulting solution was centrifuged at 3000 rpm for 10 min. Finally, 2.5 mL of the supernatant was mixed with 2.5 mL of distilled water and 0.5 mL of a 0.1% ferric chloride [FeCl_3_] solution. The absorbance of the resulting solution was measured at 700 nm using a UV-Vis spectrophotometer (Shimadzu UV-1800).

### 3.7. Inhibition of Pancreatic Lipase Activity

The inhibitory activity of the samples against pancreatic lipase was determined using a modified spectrophotometric assay based on the method described by Worsztynowicz et al. [43]. In this assay, para-nitrophenyl butyrate (pNPB) was employed as the substrate for pancreatic lipase hydrolysis. The enzymatic hydrolysis of pNPB yields para-nitrophenol, a chromogenic product that exhibits maximum absorbance at 405 nm, which was quantified using a spectrophotometric method. A pancreatic lipase stock solution (1 mg/mL) was prepared by dissolving 50 mg of porcine pancreatic lipase (Sigma-Aldrich, L3126, St. Louis, MO, USA) in 50 mL of buffer (13 mM Tris-HCl, pH 8.0, 1.3 mM CaCl_2_, and 150 mM NaCl). The solution was then centrifuged at 4000 rpm for 15 min, and the supernatant containing the enzyme was collected. A total of 50 μL of sample solution and 50 μL of pancreatic lipase solution were added to a 96-well plate and incubated at 37 °C for 10 min. Subsequently, 50 μL of 2 mg/mL pNPB was added, and the incubation continued for 20 min. The absorbance was measured at 405 nm. The percentage inhibition of pancreatic lipase activity was calculated using the following formula: inhibition rate (%) = $\left(1-\frac{A_{sample}-A_{blank}}{A_{test}-A_{control0}}\right)\times 100$.

To determine the mode of pancreatic lipase inhibition, enzyme kinetic studies were performed using varying concentrations of the substrate, para-nitrophenyl butyrate (pNPB), in the presence and absence of the test sample. Specifically, 50 μL of the sample solution at a fixed concentration of 0.2 mg/mL (or 50 μL of distilled water as a control) was mixed with 50 μL of pancreatic lipase solution at a concentration of 1 mg/mL in a 96-well microplate. The mixture was pre-incubated at 37 °C for 10 min to allow for enzyme–inhibitor interaction. Following pre-incubation, 50 μL of pNPB solutions at varying concentrations (0.2, 0.3, 0.4, 0.5, 0.6, and 0.7 mg/mL, prepared in the assay buffer) were added to the respective wells to initiate the enzymatic reaction. The reaction was allowed to proceed at 37 °C and the initial reaction rates were determined by measuring the change in absorbance at 405 nm over a defined time interval. The type of inhibition was analyzed using the Lineweaver–Burk equation [44] $\frac{1}{V}=\frac{K_{m}}{V_{max}}\left(1+\frac{\left[I\right]}{K_{i}}\right)\frac{1}{\left[S\right]}+\frac{1}{V_{max}}\left(1+\frac{\left[I\right]}{{K_{i}}^{\prime }}\right)$, where *A_sample_* is the absorbance after reaction with the sample; *A_blank_* is the absorbance of the blank; *A_test_* is the absorbance for the test; *A_control_* is the absorbance for the control. *V* and *V_max_* represent the initial reaction velocity and the maximum initial reaction velocity, respectively. *K_m_*, *K_i_*, and *K_i_′* are the Michaelis constant, competitive inhibition constant, and non-competitive inhibition constant, respectively. [*I*] is the concentration of the inhibitor and *S* is the concentration of the substrate.

### 3.8. Viability Assessment of 3T3-L1 Preadipocytes

The cytotoxicity of the test samples on 3T3-L1 preadipocytes was evaluated using a 3-(4,5-dimethylthiazol-2-yl)-2,5-diphenyltetrazolium bromide (MTT) assay. The 3T3-L1 preadipocytes were seeded in a 96-well microplate at a density of 6 × 10^3^ cells per well and allowed to adhere for 24 h in complete culture medium (DMEM supplemented with 10% FBS and 1% penicillin). Following the 24-hour adhesion period, the culture medium was replaced with fresh complete culture medium containing various concentrations of the test samples. The control group received an equivalent volume of complete culture medium without the test samples. Each concentration was tested in five replicate wells. The cells were then incubated at 37 °C in a 5% CO_2_ incubator for 24 h. After discarding the medium, the wells were washed twice with PBS. Then, 100 μg of an MTT solution was added to each well, and the cells were incubated for an additional 4 h. The medium was discarded, and 150 μL of DMSO was added to each well, followed by shaking for 15 min. The absorbance was measured at 570 nm.

### 3.9. Oil Red O Staining

The 3T3-L1 cells were rinsed five times with phosphate-buffered saline (PBS) and subsequently fixed in a 4% formaldehyde solution for 1 h. Following fixation, the cells underwent dehydration using a 60% isopropyl alcohol solution. A 0.5% Oil Red O staining solution, prepared in 60% isopropanol and diluted with distilled water at a ratio of 3:2, was filtered through a bottle-top filter (Nalgene, Thermo Fisher Scientific Inc., USA, Rochester, NY, USA) to remove any particulate matter. The filtered staining solution was then applied to the cells on the culture plate and the cells were incubated in the dark for 10 min to allow for lipid droplet staining. The stained lipid droplets within the cells were visualized using light microscopy. For quantitative analysis, the Oil Red O stain was extracted from the cells using 100% isopropanol, and the absorbance of the resulting solution, which is directly proportional to the amount of stained lipid, was measured spectrophotometrically at 520 nm [45].

### 3.10. Quantification of Intracellular Total Triglycerides (TG)

The intracellular total triglyceride (TG) content in differentiated 3T3-L1 adipocytes was quantified using a commercially available Triglyceride Assay Kit (Abcam, ab65336; Sigma-Aldrich, MAK266, St. Louis, MO, USA), with slight modifications to a method based on the work of Kang et al. [45]. Following the completion of the differentiation protocol and any treatment with the test samples, the cells cultured in 6-well plates were washed twice with ice-cold phosphate-buffered saline (PBS). The cells were then detached from the wells by trypsinization (using 0.25% trypsin-EDTA solution) and collected by centrifugation at a defined speed and duration (1500 rpm for 5 min), then a specific volume of isopropanol was added to extract the TG. The intracellular TG content was then determined using a TG assay kit.

### 3.11. RNA Extraction and Preparation for Gene Expression Analysis

Following the designated differentiation period and treatment, the cell culture medium in the 6-well plates was aspirated, and the cells were washed 3 times with phosphate-buffered saline (PBS). Subsequently, 1 mL of TRIzol reagent was added to each well and incubated on ice for 5 min to ensure complete cell lysis and RNA stabilization. The lysates were centrifuged at 12,000× *g* for 5 min at 4 °C. The resulting supernatant was transferred to a new centrifuge tube, followed by the addition of 200 μL of chloroform, and then mixed. After standing at room temperature for 5 min, the samples were centrifuged at 12,000× *g* for 15 min at 4 °C. The aqueous phase was carefully transferred to a new tube, and 600 μL of isopropanol was added and mixed. The mixture was incubated at room temperature for 10 min and then centrifuged at 12,000× *g* for 10 min at 4 °C, resulting in RNA precipitation. The supernatant was discarded, and the RNA pellet was washed with 75% ethanol, followed by centrifugation at 7500× *g* for 5 min at 4 °C. After air-drying at room temperature, the RNA pellet was resuspended in an appropriate volume of DEPC-treated water, aliquoted, and stored at −80 °C. The primer and probe sequences and concentrations were optimized according to the manufacturer’s instructions in exTaq DNA polymerase and were as follows: C/EBPα: 5′-GGGCAAAGCCAAGAAGTCGG-3′, 5′-AGCACCTTCTGTTGCGTCTC-3′, PPAR: 5′-CCATCGAGGACATCCAAGACAACC-3′, 5′-GGAGCACCTTGGCGAACAGC-3′, ACC: 5′-TTGAAGGCACAGTGAAGGCTTACG-3′, 5′-GACGCCATCTTCCTCTGTCAGTTG-3′, FAS: 5′-CTTCGCCAACTCTACCATGG-3′, 5′-TTCCACACCCATGAGCGAGT-3′, β-actin: 5′-CCACAGCTGAGAGGGAAATC-3′, and 5′-AAGGAAGGCTGGAAAAGAGC-3′.

### 3.12. Statistics Analysis

All experiments were conducted in triplicate (*n* = 3) and the results are expressed as the mean ± standard deviation (SD). Statistical analyses were performed using a one-way analysis of variance (ANOVA) followed by Duncan’s multiple range test to determine significant differences between the groups. A *p*-value < 0.05 was considered statistically significant.

## 4. Conclusions

In this investigation, a methanol extract of *P. japonicus* was prepared and subsequently fractionated using solvents of increasing polarity: hexane, ethyl acetate, and n-BuOH. The resulting fractions were exhaustively evaluated for their antioxidant potential, as assessed by DPPH radical scavenging activity and ferric reducing power, along with their inhibitory effects on pancreatic lipase. Our findings distinctly indicate that PJEA is particularly rich in phenolic compounds, exhibits substantial free radical scavenging activity, and possesses strong reducing power. Additionally, PJEA demonstrated significant biological activity by effectively inhibiting pancreatic lipase, with a half-maximal inhibitory concentration (IC_50_) of 317 μg/mL. Enzyme kinetic analyses showed that PJEA inhibits pancreatic lipase through a mixed mechanism involving both competitive and non-competitive interactions with the enzyme. Beyond its antioxidant and lipase inhibitory properties, PJEA also displayed potent anti-adipogenic activity in vitro by targeting numerous critical regulatory points in the adipocyte differentiation and lipid metabolism pathways. Specifically, PJEA significantly downregulated the expression of the master transcriptional regulators PPARγ and C/EBPα, as well as the key lipogenic enzymes FAS and ACC, in 3T3-L1 preadipocytes. This comprehensive modulation of gene expression supports the observed inhibitory effects of PJEA on adipocyte differentiation and lipid accumulation. Bioactivity-guided fractionation of the significantly active PJEA led to the successful isolation and identification of two compounds: kaempferol 3-O-α-L-rhamnoside and catechin.

While these findings provide important insights into the biological effects of *P. japonicus* extracts, a limitation of this study is the lack of detailed chromatographic profiling to confirm and quantify the comprehensive profile of phenolic and flavonoid constituents. In future research, we intend to perform HPLC–DAD–MS analysis to complement the compound isolation and to develop a robust phytochemical fingerprint for standardization purposes.

Overall, the comprehensive findings of this research provide essential data that may serve as a vital resource for the discovery and development of natural ingredients with superior radical scavenging and pancreatic lipase inhibitory activities, along with potential applications for managing obesity and related metabolic disorders.

## Figures and Tables

**Table 1:** Extraction yield, total phenolic content (TPC), total flavonoid content (TFC), and DPPH radical scavenging activity of *P. japonicus* extracts obtained with different solvents.

Solvent	Extract Yield (%)	Total Phenolic Content (mg GAE/g)	Total Flavonoid Content (mg QE/g)	DPPH Radical Scavenging Activity IC_50_ (μg/mL)
Methanol	50.12	102.6 ± 0.28 ^a,1)^	48.02 ± 1.78 ^a^	36.33 ± 0.74 ^a^
Ethanol	38.67	83.19 ± 2.96 ^c^	19.36 ± 1.01 ^c^	46.12 ± 1.75 ^c^
Water	48.34	96.71 ± 3.63 ^b^	23.57 ± 0.64 ^b^	40.47 ± 2.14 ^b^
Acetone	29.31	62.36 ± 0.94 ^d^	16.67 ± 0.21 ^d^	70.51 ± 0.96 ^d^
BHA	ND ^1)^	ND	ND	6.85 ± 1.95

^1)^: Not detected. Values are expressed as mean ± SD from three independent experiments (*n* = 3). There were no significant differences (*p* < 0.05) between means in the same column, according to Duncan’s LSR test.

**Table 2:** Antioxidant activity of extract and solvent fractions from *P. japonicus*.

Extraction	DPPH Radical Scavenging Activity IC_50_ (μg/mL)	Total Phenolic (mg GAE/g)	Total Flavonoid (mg QE/g)
MeOH	36.33 ± 2.81 ^b,1)^	102.60 ± 4.58 ^b^	48.02 ± 1.78 ^b^
Hexane	108.74 ± 3.16 ^d^	31.02 ± 3.26 ^d^	13.67 ± 2.31 ^d^
EtOAc	5.39 ± 1.08 ^a^	198.51 ± 0.63 ^a^	68.63 ± 0.37 ^a^
*n*-BuOH	65.31 ± 3.76 ^c^	96.50 ± 3.79 ^c^	22.35 ± 1.33 ^c^
Water	123.87 ± 2.85 ^e^	2.98 ± 0.88 ^e^	2.18 ± 1.46 ^e^
BHA	6.85 ± 1.95	ND ^1)^	ND

^1)^: Not detected. Values are expressed as mean ± SD from three independent experiments (*n* = 3). There were no significant differences (*p* < 0.05) between means in the same column, according to Duncan’s LSR test.

## Data Availability

The data presented in this study are contained within the article.
